# Drunkenness and Crime
*Proceedings of the Society for the Study of Inebriety. Annual address by the President, Dr. Norman Kerr. (London: H. K. Lewis, 136, Gower Street, W.C.) Sixpence.


**Published:** 1892-04-09

**Authors:** 


### DRUNKENNESS AND CRIME.*

Dr. Norman Kerr's presidential address consists in large measure of a recapitulation of the verdicts and sentences in recent criminal cases, where the accused has been either drunk when the crime was committed, or his mental condition so effected by inebriety, either his own or his parents, as to render him, in Dr. Kerr's opinion, irresponsible for his actions. It is needless to say that this view is not invariably held by our judges, but Dr. Kerr notes with satisfaction an increasing tendency to act upon it. Of course, the results of this course of proceeding are not wholly satisfactory. Dr. Kerr expresses his approval of the judgment, in the Edinburgh Court of Session, of Lord Young, who decided that there was no case against a woman accused of culpable homicide, who, being a confirmed inebriate, allowed her child, aged six weeks, to die of starvation and neglect. But in such a case mere acquittal, however legally and even morally just, does little either for the criminal or for the possible victims. Any modification of the punishment of crimes due to drunkenness must be accompanied by such a change in the law as will empower judges to treat drunkards as what they are—invalids and lunatics. Dr. Kerr highly and justly eulogises the conduct of Sir Peter Edlin, who, in a case of inebriate kleptomania which was brought before him, acquitted the prisoner, but exacted from him a promise to enter a home for inebriates. If it were always within the power of judge or magistrate to consign inebriate criminals to a suitable hospital, with a view to either cure or life-long detention, no one could desire a better thing; and it is this which Dr. Kerr would fain see the Legislature enact. Our present system of voluntary retreats is to a large extent a failure. Very few inebriates or narcomaniacs—perhaps those addicted to opium, chloral, or other drugs less than those who are given to alcohol—have the moral courage to shut themselves up against temptation, and at present no one can compel them to such a step. In any case, these retreats are available only for those who themselves, or through their friends, can pay for treatment. To the poorer inebriate there is only the magistrate's tribunal, and the ever-recurring, futile sentence of a few days' imprisonment, useless for either punishment or reform. When a woman has been sent to prison fifty-two times in one year, or when another at the age of thirty-five records 700 convictions, it is time to admit that something more than a brief incarceration is required. One cannot but agree with Dr. Norman Kerr that "frequent convictions for drunkenness are *prima facie* evidence of a diseased state of the prisoner's brain," and accepting as true also his statement that alcoholic indulgence, by causing hypertrophy of the connective tissue, develops "a permanent lesion of the brain, as clearly defined as an abrasion of the skin or a broken arm," we must desire for criminal inebriates a treatment similar to that meted out to other criminal lunatics. That must be done by the Legislature; but the society of which Dr. Norman Kerr is the energetic chief is doing much to bring about a state of public feeling which must sooner or later find voice in a legal enactment.

* Proceedings of the Society for the Study of Inebriety. Annual address by the President, Dr. Norman Kerr. (London: H. K. Lewis, 136, Gower Street, W.C.) Sixpence.

